# Cigarette smoke promotes dendritic cell accumulation in COPD; a Lung Tissue Research Consortium study

**DOI:** 10.1186/1465-9921-11-45

**Published:** 2010-04-26

**Authors:** Robert Vassallo, Paula R Walters, Jeffrey Lamont, Theodore J Kottom, Eunhee S Yi, Andrew H Limper

**Affiliations:** 1 The Thoracic Diseases Research Unit, Division of Pulmonary Critical Care, Department of Internal Medicine, the Clinical Immunology and Immunotherapeutics Program, Mayo Clinic and Foundation, Rochester, Minnesota, 55905, USA; 2 The Division of Anatomic Pathology, Mayo Clinic and Foundation, Rochester, Minnesota, 55905, USA; 3 The Department of Biochemistry and Molecular Biology, Mayo Clinic and Foundation, Rochester, Minnesota, 55905, USA

## Abstract

**Background:**

Abnormal immune responses are believed to be highly relevant in the pathogenesis of chronic obstructive pulmonary disease (COPD). Dendritic cells provide a critical checkpoint for immunity by their capacity to both induce and suppress immunity. Although evident that cigarette smoke, the primary cause of COPD, significantly influences dendritic cell functions, little is known about the roles of dendritic cells in the pathogenesis of COPD.

**Methods:**

The extent of dendritic cell infiltration in COPD tissue specimens was determined using immunohistochemical localization of CD83+ cells (marker of matured myeloid dendritic cells), and CD1a+ cells (Langerhans cells). The extent of tissue infiltration with Langerhans cells was also determined by the relative expression of the CD207 gene in COPD *versus *control tissues. To determine mechanisms by which dendritic cells accumulate in COPD, complimentary studies were conducted using monocyte-derived human dendritic cells exposed to cigarette smoke extract (CSE), and dendritic cells extracted from mice chronically exposed to cigarette smoke.

**Results:**

In human COPD lung tissue, we detected a significant increase in the total number of CD83+ cells, and significantly higher amounts of CD207 mRNA when compared with control tissue. Human monocyte-derived dendritic cells exposed to CSE (0.1-2%) exhibited enhanced survival *in vitro *when compared with control dendritic cells. Murine dendritic cells extracted from mice exposed to cigarette smoke for 4 weeks, also demonstrated enhanced survival compared to dendritic cells extracted from control mice. Acute exposure of human dendritic cells to CSE induced the cellular pro-survival proteins heme-oxygenase-1 (HO-1), and B cell lymphoma leukemia-x(L) (Bcl-xL), predominantly through oxidative stress. Although activated human dendritic cells conditioned with CSE expressed diminished migratory CCR7 expression, their migration towards the CCR7 ligand CCL21 was not impaired.

**Conclusions:**

These data indicate that COPD is associated with increased numbers of cells bearing markers associated with Langerhans cells and mature dendritic cells, and that cigarette smoke promotes survival signals and augments survival of dendritic cells. Although CSE suppressed dendritic cell CCR7 expression, migration towards a CCR7 ligand was not diminished, suggesting that reduced CCR7-dependent migration is unlikely to be an important mechanism for dendritic cell retention in the lungs of smokers with COPD.

## Introduction

Chronic obstructive pulmonary disease (COPD) is a slowly progressive destructive lung disease induced primarily by cigarette smoking in developed countries [1]. Although COPD is associated with significant abnormalities in local immunity, the precise roles of inflammation, and the distinct roles of innate and acquired immune cells in the pathogenesis of COPD remain incompletely characterized [2]. Among the immune cell types infiltrating the COPD airways, the extent of CD8 T cell, and small airway dendritic cell infiltration correlate with COPD severity as determined by lung function testing, suggesting that these immune cells play important roles in the pathogenesis [3].

Dendritic cells are rare but critical immune cells that are distributed in sub-epithelial, interstitial and pleural compartments, where they usually exist as immature antigen presenting cells [4]. At least three major phenotypic and functional subsets have been described; classic or conventional myeloid dendritic cells, epithelial-associated CD1a positive dendritic cells (analogous to epithelial associated Langerhans cells in the skin), and plasmacytoid dendritic cells [4,5]. The effect of smoking on dendritic cell profusion and activation in COPD is somewhat controversial with recent studies showing conflicting findings [3,6,7]. Demedts and colleagues reported that the accumulation of CD207-positive dendritic cells (Langerhans cells) in the epithelium and adventitia of small airways in COPD was greater than that occurring in never-smokers or smokers without COPD [3]. Demedts et al also reported that the number of Langerhans cells in the small airways of COPD patients increase in proportion with disease severity, suggesting that these abnormally accumulated Langerhans cells may directly participate in the pathogenesis of COPD [3]. In contrast, another study reported no difference in the numbers of Langerhans cells in the airway biopsies of smokers with COPD when compared to ex-smokers or non-smokers without COPD [6].

The current study was designed to determine whether COPD is associated with increased numbers of Langerhans-type dendritic cells (defined by surface expression of CD1a, or the presence of transcripts for the Langerhans cell restricted gene CD207), or matured dendritic cells (defined by surface CD83 expression), utilizing human COPD lung tissue procured through the Lung Tissue Research Consortium (LTRC). In addition, complimentary studies were conducted using relevant *in vitro *human and *in vivo *murine models to determine mechanisms by which cigarette smoke constituents promote dendritic cell persistence in the lung. Specifically, we sought to determine whether cigarette smoke promotes dendritic cell retention in the lung by promoting dendritic cell survival. In addition, we sought to determine the effect of cigarette smoke constituents on key migratory chemokine expression and migratory capacity of dendritic cells.

## Methods

### Immunohistochemical detection of dendritic cells in COPD tissue

Slides of formalin-fixed, paraffin-embedded specimens were obtained from twenty-four patients - 8 controls (these control subjects would have been classified as GOLD stage 0, or at risk subjects based on the 2001 GOLD guidelines [8], but would now be classified as not having spirometric criteria of COPD based on a post-bronchodilator FEV_1_/FVC ratio > 70% and % predicted FEV1 > 80; 1 subject had an FEV1 of 78% predicted but an FEV_1_/FVC ratio > 70% [9]), 8 with moderate (post-bronchodilator FEV_1_/FVC ratio < 70%; FEV_1 _50-80% predicted), and 8 with severe COPD (post-bronchodilator FEV_1_/FVC ratio < 70%, FEV_1 _< 50%) based on spirometry. All tissue samples were obtained from the National Institutes of Health funded Lung Tissue Research Consortium (LTRC: http://www.ltrcpublic.com - a detailed account of the LTRC protocol used is available to the public at http://www.ltrcpublic.com/PRO_NOV_2009.pdf). Asthma and other causes of obstructive lung disease were excluded in all subjects based on history, physical examination, and spirometric data. Tissue sections were de-paraffinized in 3 changes of xylene (5 minutes each), hydrated in 2 changes of 100% ethanol (10 minutes each), and subsequently hydrated in 2 changes of 95% ethanol (10 minutes each). Antigen unmasking was performed in a pressure cooker and with 10 mM sodium citrate buffer, pH 6.0. Endogenous peroxidase activity was quenched by placing slides in a 1% hydrogen peroxide solution in methanol for 30 minutes. Tissue slides were then washed (1× Dako Wash Buffer) and immunostaining performed using a commercially available VectaStain Elite ABC (Vector Labs) kit according to the manufacturer's instructions. Tissues were stained using CD1a (Dako; marker for Langerhans cells) and CD83 (Dako; marker for mature dendritic cells). Images of the stained tissue were digitally captured at a 10× magnification using a NanoZoomer system (Bacus laboratories, Olympus America). The percent area of lung tissue positively stained for CD1a and CD83 were quantitatively determined using IHCscore software (Bacus lab, Olympus America), as recently described [10]. Image capture and quantitative determination of tissue staining for CD1a and CD83 were performed by one the investigators (JL) in a blinded manner (control vs COPD). The area of tissue positively stained was estimated relative to the area of total lung tissue present on the slide to determine the extent of Langerhans cell and mature dendritic cell infiltration for each sample. Relevant demographic and physiologic lung function variables for the study population were obtained from LTRC. Correlations were performed between quantitative surface marker expression (CD1a and CD83) and cumulative tobacco exposure (pack years of cigarette smoked), as well as physiologic lung function measurements.

### Determination of CD207 and Osteopontin gene expression levels in COPD and control tissues

Quantitative real-time RT-PCR was performed to determine the relative expression of Langerhans cell restricted CD207 (langerin) gene transcripts in COPD and control tissues. For qPCR analysis, the CFX96 Real-Time PCR detection System was utilized (Bio-Rad). RNA was extracted from whole lung frozen tissue using a Qiagen RNA isolation kit. RNA was extracted only from frozen tissue specimens, and all extractions were performed in batch format in the institutional Advanced Genomics Technology Center. Samples with an RNA Integrity Number (RIN: determined by Agilent Bioanalyzer software) < 7.5 were excluded from the analysis. To make cDNA for qPCR analysis, SuperScript^® ^III Reverse Transcriptase was employed (Invitrogen). To quantify the final cDNA PCR products, SYBR^® ^Green PCR Master Mix was used. Conditions for the PCR reactions were as follows: 50°C for 2 min, 95°C for 2 min, followed by 40 cycles of 95°C for 15 sec, 60°C for 30 sec, and 72°C for 30 sec. During the 72°C temperature, analysis of the SYBR fluorophore for quantification was conducted. Relative expression levels of CD207 mRNA were calculated by normalizing to the level of GAPDH mRNA by using comparative threshold cycle (ct) method, in which fold difference = 2-(Δct of target gene-Δct of reference). Primers for amplification of CD207 mRNA were 5'-GTGGACAAACAGAACATCTCCCTC-3' and 5'-GACCTTTCAGCAACTGGACATTG-3'. GAPDH transcript was amplified with primers 5'-CGGTATTTGGTCGTATTGGGC-3' and 5'-TGGAAGATGGTGATGGGATTTC-3'. An identical approach was used to determine the relative expression levels of osteopontin mRNA using the following primers: 5'-GCAGTGATTTGCTTTTGCCTCC-3' and 5'-CTTTCGTTGGACTTACTTGGAAGG-3'.

### Generation of cigarette smoke extract (CSE)

For *in vitro *studies, aqueous CSE was prepared from Kentucky research cigarettes 3RF4 as recently described [11]. The nicotine levels in the CSE preparations were measured in the Mayo institutional clinical laboratory using liquid chromatography-tandem mass spectrometry: the nicotine content measured in 1% CSE was 174 ng/ml. Cellular experiments were conducted with CSE concentrations <3% because of viability studies that demonstrated cellular toxicity as determined by the XTT assay and AnnexinV/propridium iodide staining with CSE preparations >3%.

### Human monocyte-derived dendritic cells

Human monocytes were isolated from buffy coats obtained from healthy non-smoking adult blood donors, and monocyte-derived dendritic cells were generated using a 6 day differentiation protocol with recombinant human IL-4 and GM-CSF as previously described [11,12]. Maturation was induced by overnight culture with 100 ng/ml lipopolysaccharide [LPS from E. coli; Sigma] unless otherwise specified.

### Determination of cellular protein levels by immunoblotting

Semi-quantitative determination of cellular protein levels were obtained by immunoblotting as recently described [12]. Human monocyte-derived dendritic cells were plated at a concentration of 1 × 10^6^/ml in complete media [RPMI, 10% fetal bovine serum] and GM-CSF/IL-4 as previously described [12]. Cigarette smoke extract and LPS were added to the cells at the time points indicated. Protein lysates were prepared using RIPA buffer [150 mM NaCl, 1.0% Igepal CA-630, 0.5% sodium deoxycholate, 0.1% SDS, 50 mM Tris] as recently described [12]. Antibodies used in immunoblotting included Bcl-xL (Cell Signaling # 2762), HO-1 (Stressgen # SPA-896), and CCR7 (eBioscience # 14-9977-82).

### Determination of dendritic cell migration

Human monocyte-derived dendritic cells were treated for 18 hours with 100 ng/ml of LPS and 50 ng/ml of IFN-γ, with or without 2% CSE, or 2% CSE and 1 mM NAC. After this period of activation, dendritic cells were washed in RPMI, and equal numbers (0.5 × 10^6^) were placed in 0.5 ml of RPMI without serum and assayed for migration towards CCL21 placed in the lower chamber of a transwell plate. The lower chambers of Transwell plates (5.0-μm pore size; Corning, Acton, MA) were filled with 0.5 ml of complete media (RPMI with 10% fetal calf serum) supplemented with 10 ng/ml CCL21. Human dendritic cells placed in the upper chambers of the Transwell plates were allowed to migrate to the lower chamber for 4 hours at 37°C in 5% CO2. The total numbers of migrated dendritic cells from the lower chambers were determined by flow cytometry (60-second counts).

### *In vivo *exposure of mice to cigarette smoke

All murine experiments described were approved by the institutional animal use and care committee. To further expand on our *in vitro *studies, we conducted *in vivo *studies to determine the effect of chronic cigarette smoking on lung and systemic dendritic cell survival. Mice were chronically exposed to cigarette smoke generated from a Teague TE-2 system, as recently described [12]. This smoking chamber is a manually-controlled cigarette smoking machine that produces a combination of side-stream and mainstream cigarette smoke in a chamber, which is then transported to a collecting and mixing chamber where varying amounts of air is mixed with the smoke mixture. In this model, mice were exposed to regulated concentrations of cigarette smoke generated from 2 cigarettes every 10 minutes for a total of 3 hours/day, 5 days/week for a total of 4-6 weeks. Following 4-6 weeks in the smoking chamber, mice were sacrificed, blood was removed by right heart puncture and submitted for nicotine analyses, and dendritic cells from lung and splenic tissues isolated as recently described [12].

### Statistical Analysis

Statistical differences were considered to be significant if P was <0.05. For the lung tissue analysis, data is expressed as means and comparisons between means were performed using the Mann Whitney U test. Correlations between lung tissue characteristics and clinical parameters were performed using the Spearman test. For the *in vitro *studies with dendritic cells, data is expressed as means and standard error, while comparison between multiple means was done with ANOVA. Statistical analysis was performed using SigmaPlot5. Densitometry was performed using Image J software http://rsbweb.nih.gov/ij/.

## Results

### Dendritic cells accumulate in COPD lungs

**Figure 1:**
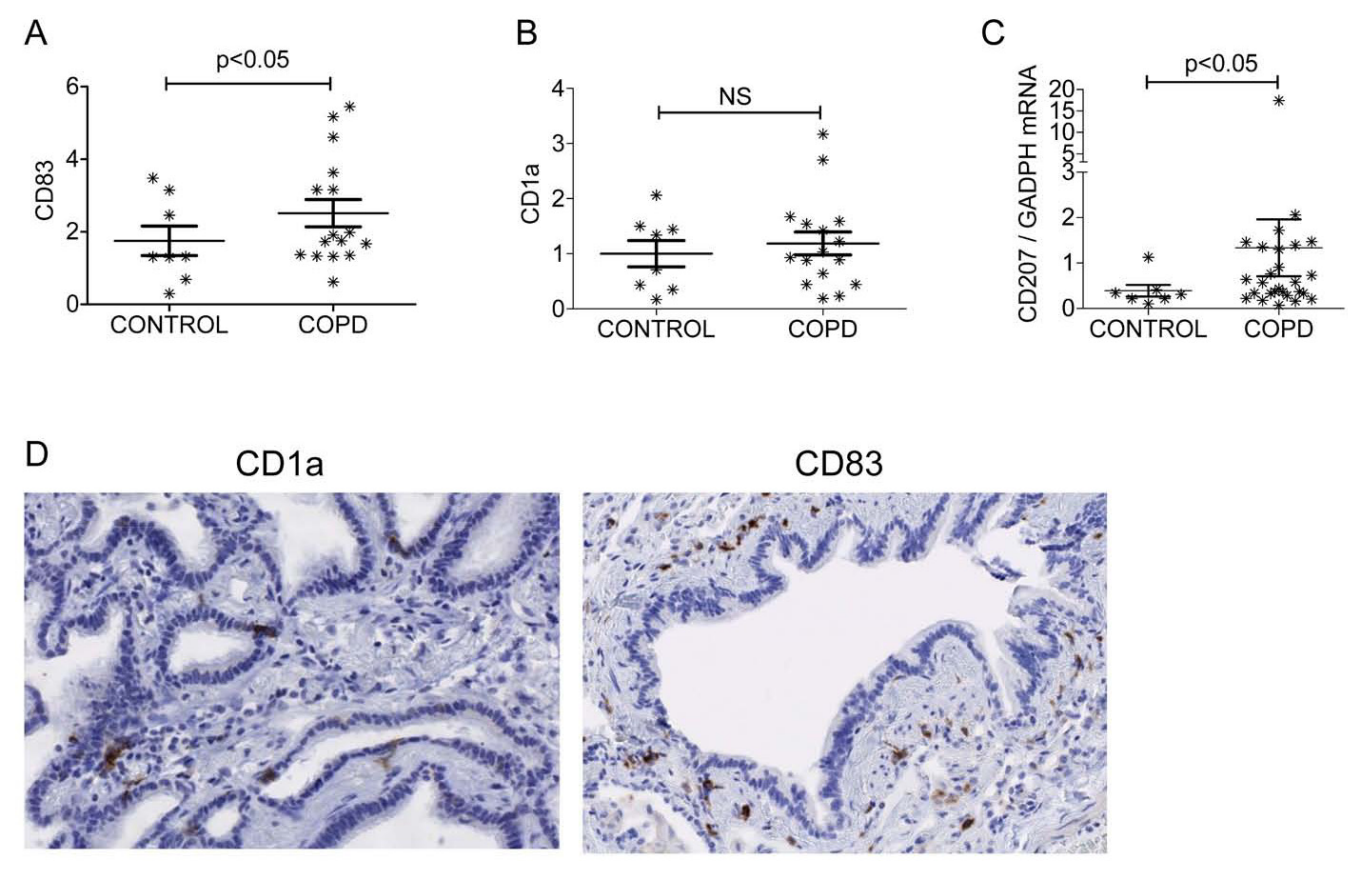
**COPD tissue contains increased numbers of CD83+ cells and CD207 (Langerin) mRNA levels compared to control tissue**. **A) **Quantitative total tissue expression of CD1a+ cells identified in whole tissue slides from 8 controls and 16 COPD subjects. The values on the vertical axis refer to the area of positive staining relative to the entire scanned tissue biopsy. Graph shows mean and SEM of all data points. NS = not significant. **B) **Quantitative total tissue expression of CD83+ cells in 8 controls and 16 COPD subjects. The values on the vertical axis refer to the area of positive staining relative to the entire scanned tissue biopsy. Graph shows mean and SEM of all data points. **C) **Increased CD207 mRNA expression in total lung tissue from patients with COPD (n = 27) compared with controls without COPD (n = 7). Relative CD207 mRNA expression is shown as the ratio of the number of transcripts for CD207 to the number of transcripts for the housekeeping gene GAPDH. Graph shows mean and SEM of all data points. **D) **Representative images demonstrating the dendritic cells with dark brown membranous positivity on CD1a (left panel) and CD83 (right panel) stainings. Right panel; 58-year old ex-smoker with FEV1 63% predicted. Left panel; 78-year old ex-smoker with FEV1 60% predicted.

Accumulation of matured myeloid dendritic cells (defined as CD83+), or Langerhans-type dendritic cells (defined as CD1a+) in lung biopsy specimens, was evaluated in 24 subjects from the LTRC database. Subject characteristics are shown in Table 1. As expected, the % predicted FEV_1 _and FEV_1_/FVC ratio were significantly lower in patients with COPD when compared with control subjects. Rather than count specific numbers of cells, quantitative measures of expression for the dendritic cell receptors, CD83 and CD1a, were estimated from electronically scanned entire control and COPD tissue slides, and the relative intensity of expression for either marker were determined using image capture analysis. A statistically significant increase in CD83+ staining cells - a cell surface receptor expressed by matured dendritic cells - was detected in COPD tissues compared to control tissues (Figure 1A, Mann Whitney U p = 0.049). The extent of CD83+ staining correlated with cumulative tobacco consumption defined by pack years of cigarettes smoked (R 0.398, p = 0.026), but did not correlate with % predicted FEV_1 _(R -0.194, p = 0.182) or % predicted DLCO (R -0.084, p = 0.348). The extent of CD83 staining on tissue slides also correlated with intensity of CD1a expression (R 0.566, p = 0.002). Quantitative total tissue expression of CD1a did not show a statistically significant difference between control and COPD tissue (Figure 1B, Mann Whitney U p = 0.301). The extent of CD1a staining on tissue slides did not correlate with cumulative pack years smoked (R 0.305, p = 0.073) or % predicted FEV_1 _(R -0.241, p = 0.128), but inversely correlated with the % predicted DLCO (R -0.350, p = 0.047).

**Table 1:** Demographic and pulmonary function characteristics of 16 COPD patients and 8 control subjects from whose lung biopsies were used for immunohistochemical detection of CD1a+ Langerhans cells and CD83+ mature dendritic cells.

	Controls	COPD	P value
Gender M:F	2:6	9:7	
Age - median (range)	63.5 (56-78)	65.5 (49-78)	0.779
Smoking history (pack years)			
Median (range)	50.0 (2-110)	51.5 (5-180)	0.625
FEV1 (% predicted)			
Median (range)	88.0 (78-107)	43.5 (27-75)	<0.001
FEV1/FVC - Median	101.5	59.7	<0.001
DLCO (% predicted)			
Median (range)	62.0 (34-122)	54.0 (27-99)	0.416

As the extent of CD1a positive staining in COPD tissue appeared to show a higher trend in comparison with control tissue, we determined the relative amounts of CD207 mRNA as an alternative approach to determine whether smokers with COPD have increased numbers of Langerhans cells infiltrating lung tissue. To determine this, RNA was extracted from 12 frozen lung biopsy samples available from the tissue samples used in the immunohistochemical approach described above (the remaining samples could not be used as the extracted RNA was not of adequate quality), and an additional 22 frozen lung samples obtained through the LTRC. All samples used in this analysis had RIN > 7.5. The 34 samples analyzed included 7 controls without COPD (mean % predicted FEV_1 _91.3, range 84-103) and 27 COPD subjects (mean % predicted FEV_1 _38.3, range 22-75). All patients were either current or former smokers. A statistically significant increase in CD207 mRNA was found in lung tissue with COPD compared to lung tissue without COPD; Figure 1C, mean/SE CD207 expression 1.335 ± 0.627 vs 0.389 ± 0.128 respectively; p = 0.048.

Recent studies have shown that cigarette smoke induces osteopontin production by lung epithelial cells and macrophages [13,14]. Osteopontin is involved in the regulation of Th-1 immunity and autoimmunity, is chemotactic and anti-apoptotic to dendritic cells, and when over-expressed in murine lungs, induces dendritic cell recruitment [15,16]. Analysis of osteopontin mRNA levels in the 7 control and 27 COPD tissues used to determine CD207 mRNA levels, did not show a significant difference between controls and COPD (0.188 ± 0.14 vs 0.297 ± 0.15 respectively, p = 0.17). Although osteopontin mRNA levels were not significantly different between the control and COPD groups, osteopontin mRNA levels in the 27 tissue specimens with COPD correlated with CD207 mRNA (R = 0.6529, p = 0.001). Taken together these data indicate that COPD lung tissue is infiltrated by greater numbers of both cells expressing the Langerhans cell gene CD207, and matured CD83+ dendritic cells. The correlation between osteopontin and CD207 mRNA levels suggests a potential link between osteopontin regulation and Langerhans cell infiltration in COPD.

### Cigarette smoke extract and cigarette smoking promote dendritic cell survival

**Figure 2:**
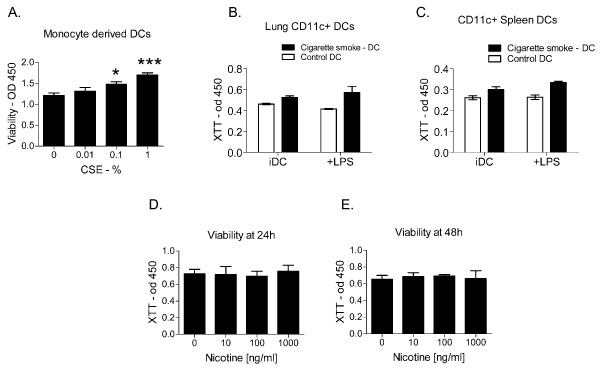
**Cigarette smoke extract and cigarette smoking promote dendritic cell survival**. **A) **Human dendritic cells (1 × 10^6^/ml in complete media) were incubated in the presence of absence of CSE (0.01 to 1%) for 24 hours prior to determination of cellular viability using the XTT assay. *p < 0.05 and ***p < 0.001 compared to control (Dunnett's test). Data shown is representative of four independent experiments. **B) **Lung CD11c+ dendritic cells were extracted from mice exposed to cigarette smoke for ≥4 weeks in a smoking chamber and adjusted to a concentration of 1 × 10^6^/ml in media containing 5 ng/ml of murine GM-CSF. Viability of immature and LPS-matured lung dendritic cells from 4 cigarette smoke exposed mice and 4 control mice was performed using XTT assay following 24 hours of incubation. iDC = immature DC. 2-way ANOVA p < 0.01. **C) **Spleen CD11c+ dendritic cells were also extracted from mice exposed to cigarette smoke for ≥4 weeks in a smoking chamber and adjusted to a concentration of 1 × 10^6^/ml in media containing 5 ng/ml of murine GM-CSF. Viability of immature and LPS-matured spleen dendritic cells was also performed using XTT assay following 24 hours of incubation. Data shown in B and C are representative of 2 independent experiments. **D **and **E) **Human dendritic cells (1 × 10^6^/ml in complete media) were incubated with 0-1000 ng/ml nicotine for 24 or 48 hours prior to determination of viability with an XTT assay. Data shown in D and E are representative of 3 independent experiments. Data shown are means ± SEM. *p < 0.05, **p < 0.01.

We speculated that enhanced dendritic cell survival may be a potential mechanism by which the observed increase in dendritic cell numbers occurs in COPD. To determine whether cigarette smoke promotes dendritic cell survival, immature human monocyte-derived dendritic cells were generated and subsequently incubated with increasing concentrations of freshly generated CSE (0.01 to 1%). After 24 hours of incubation in a standard tissue culture chamber, cellular viability was determined using the XTT assay. A statistically significant increase in cellular viability was observed when dendritic cells were incubated with CSE concentrations >0.1% (Figure 2A, one-way ANOVA p = 0.001; Dunnett's post-test for 0.1% CSE vs 0 and 1% CSE vs O = <0.05 and <0.001 respectively). To further characterize whether cigarette smoke promotes dendritic cell survival, we conducted complimentary studies on lung and splenic dendritic cells (defined as CD11c+ cells) extracted from mice exposed to cigarette smoke for ≥4 weeks in a Teague smoking chamber as previously described [11,12]. Lung and spleen dendritic cells were purified by magnetic sorting [12], and adjusted to a concentration of 1 × 10^6^/ml in media containing 5 ng/ml of murine GM-CSF. Viability of immature and LPS-matured lung and splenic dendritic cells from cigarette smoke exposed mice and control mice was performed using XTT assay following 24 hours of incubation in a culture chamber. Consistent with the observed effect on human dendritic cells, a statistically significant increase in viability was observed in both murine lung and systemic (spleen) dendritic cells from cigarette smoke exposed mice, in comparison with control mice (Figure 2B; 2-way ANOVA p = 0.007 for smoking effect: Figure 2C; 2-way ANOVA p < 0.001 for smoking effect). Thus, chronic exposure to cigarette smoke *in vivo *enhances *ex vivo *survival of both immature and LPS-matured lung and systemic dendritic cells. Cigarette smoke and CSE contain thousands of chemicals: to determine whether the nicotine component of cigarette smoke or CSE enhances dendritic cell survival *in vitro*, human dendritic cells were incubated with nicotine for up to 48 hours and viability measured with the XTT assay. Incubation of dendritic cells with nicotine concentrations that include those described in the circulation of active smokers [17,18], and those measured in CSE, failed to augment dendritic cell survival *in vitro *(Figure D and E, p < 0.05 by ANOVA).

### Cigarette smoke extract induces dendritic cell survival proteins

**Figure 3:**
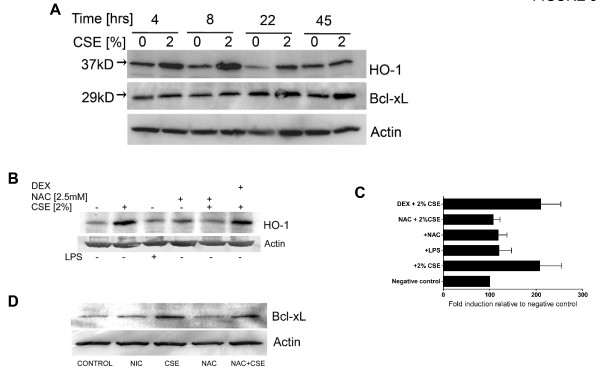
**Cigarette smoke extract induces dendritic cell survival proteins**. **A) **Human dendritic cells (1 × 10^6^/ml complete media) were incubated with 2% CSE and cellular lysates prepared following 4, 8, 22 and 45 hours. Equal amounts of protein from whole cell lysates were electrophoretically separated, transferred to nitrocellulose, and analyzed for HO-1 and Bcl-xL protein using relevant antibodies as described in methods. β-Actin protein levels were determined as an internal control to ensure equal protein loading. Representative of two independent experiments. **B) **Human dendritic cells (1 × 10^6^/ml) were incubated with complete media in the presence of absence of freshly-prepared 2% CSE, and in the presence or absence of either 2.5 mM NAC as an inhibitor of oxidative stress, or 10 μg/ml dexamethasone (NAC and dexamethasone where added 60 minutes prior to stimulation with CSE). Following 6 hours, whole cell lysates were prepared, and 50 μg of total protein were separated on a 12% gel and immunoblotting performed for HO-1. Representative of 3 independent experiments. **C) **Pooled data from 3 independent experiments performed as outlined in 3B is summarized in graph format. Actin protein levels were determined as an internal control and densitometry was performed [ratio of HO-1: Actin determined] using Image J software. The negative control samples (prepared from unstimulated dendritic cells) was arbitrarily assigned a 100% value. **D) **Human dendritic cells (1 × 10^6^/ml) were incubated with complete media in the presence of absence of freshly-prepared 2% CSE or 1000 ng/ml of nicotine. In other wells, 2.5 mM NAC was added as an inhibitor of oxidative stress. Following 48 hours, whole cell lysates were prepared, and 50 μg of total protein were separated on a 12% gel and immunoblotting performed for Bcl-xL. Representative of 2 independent experiments.

The observation that both CSE and cigarette smoke exposure enhance dendritic cell survival, led us to speculate that cigarette smoke constituents activate pro-survival or anti-apoptotic factors in dendritic cells. To test this, human dendritic cells were incubated with CSE, and cellular levels of known pro-survival factors were determined using immunoblotting. Following variable periods of incubation with CSE, protein lysates were prepared, and the cellular levels of 2 key cell survival proteins previously described to be activated in smokers macrophages [19] - heme-oxygenase-1 (HO-1), and Bcl-xL - were determined. Figure 3A shows the induction of both HO-1 and Bcl-xL protein levels in whole cell lysates of dendritic cells incubated with CSE. The induction of HO-1, an inducible cytoprotective cellular sensor of oxidative stress, occurs early and peaks by 8 hours following acute stimulation. In contrast, the induction of Bcl-xL, an anti-apoptotic member of the bcl family of proteins that promotes dendritic cell survival *in vitro *[20] and *in vivo *[21], accumulates after 24-48 hours of incubation with CSE (Figure 3A). To determine whether cigarette smoke induced oxidative stress is responsible for induction of dendritic cell HO-1, human dendritic cells (1 × 10^6^/ml media) were incubated with complete media in the presence or absence of freshly-prepared 2% CSE, and in the presence or absence of either 2.5 mM NAC as an inhibitor of oxidative stress, or 10 μg/ml dexamethasone. NAC and dexamethasone where added 60 minutes prior to stimulation with CSE. Following 6 hours of incubation with CSE and relevant inhibitors of oxidative stress (NAC) or inflammatory gene transcription (dexamethasone), whole cell lysates were prepared, and equal amounts of protein from each sample (50 μg) were separated on a 12% gel and immunoblotting performed for HO-1. As illustrated in Figures 3B and 3C, CSE-induced upregulation of HO-1 was completely suppressed by NAC, but unaffected by dexamethasone, implicating oxidative stress as the most likely inducer of HO-1 protein in cigarette smoke stimulated dendritic cells. In parallel experiments, we determined whether induction of Bcl-xL protein by CSE was primarily induced by oxidative constituents. To determine this, whole cell protein lysates were prepared from human dendritic cells incubated in the presence or absence of 2.5 mM NAC and stimulated with 2% CSE for 48 hours. As shown in Figure 3D, whereas CSE induced Bcl-xL protein, nicotine had virtually no effect on endogenous Bcl-xL levels. Dendritic cells pre-incubated with NAC failed to upregulate Bcl-xL protein levels, indicating oxidative stress as a predominant mechanism by which CSE induces Bcl-xL protein in dendritic cells.

### Oxidative cigarette smoke constituents suppress dendritic cell CCR7 expression but do not suppress dendritic cell migration

**Figure 4:**
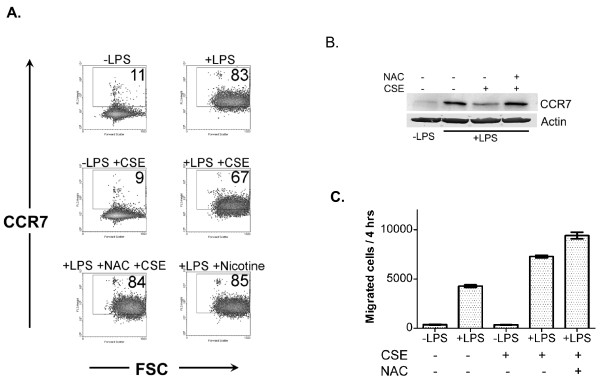
**Cigarette smoke extract inhibits CCR7 expression but does not suppress migration of dendritic cells towards a CCR7 ligand**. **A) **Human dendritic cells were incubated for 18 hours in the presence or absence of 1% CSE. The NAC group was pretreated with 1 mM NAC for 60 minutes prior to the addition of 1% CSE and LPS. The nicotine group was pretreated with 1000 ng/ml nicotine for 60 minutes prior to the addition of LPS. Surface CCR7 expression was subsequently determined with flow cytometry. The numbers in the quadrants refer to the percentage of cells in that quadrant. Data shown is representative of 3 independent experiments. **B) **Human dendritic cells (1 × 10^6^/ml in 5 ml complete media) were incubated overnight with or without 2% CSE, or 1 mM NAC. Whole cellular CCR7 protein levels were determined following 18 hours using immunoblotting. β-Actin protein levels were determined as an internal control to ensure equal protein loading. Data shown is representative of 3 independent experiments. **C) **Half a million dendritic cells under different conditions suspended in 0.5 ml RPMI without serum, placed in cell culture inserts, and inserted into wells containing 0.5 ml of RPMI with 10% fetal calf serum and 10 ng/ml of CCL21. The number of migrated cells in the lower chamber was determined using flow cytometry. Data shown Data shown are means ± SEM. Data shown is representative of 4 independent experiments.

In addition to augmented survival, we speculated that cigarette smoke may promote retention and accumulation of dendritic cells in the lung by diminishing migration to secondary lymph nodes. We focused our analysis on CCR7 expression, since deficiency of CCR7 results in impaired or absent migration of dendritic cells in mice [22-24], and CCR7 was previously reported to be suppressed in lung dendritic cells of smokers with COPD [25]. The expression of CCR7 by immature and LPS-matured human monocyte-derived dendritic cells was determined following overnight incubation of dendritic cells cultured in the presence of either 2% CSE or 1000 ng/ml nicotine. To determine whether CSE-induced oxidative stress mediates suppression of CCR7 following LPS activation, a control group of dendritic cells were pretreated with 1 mM NAC for 60 minutes prior to the addition of 2% CSE and LPS. As expected, LPS robustly induced the expression of surface CCR7 (Figure 4A). The addition of 2% CSE to the culture media, but not 1000 ng/ml nicotine, resulted in significant reduction of surface CCR7 expression (Figure 4A). Pre-incubation of dendritic cells with NAC prior to the addition of CSE resulted in complete abrogation of the inhibitory effect of CSE on LPS-mediated dendritic cell CCR7 expression, implying that oxidative stress is responsible for suppression of CCR7 (Figure 4A). Identical findings were observed when the effect of CSE on whole cellular CCR7 levels was determined using immunoblotting (Figure 4B). In parallel to the flow cytometric findings, dendritic cells incubated overnight with CSE and LPS had substantially lower cellular levels of CCR7 compared to dendritic cells activated with LPS alone (Figure 4B). In addition, pre-incubation of dendritic cells with 1 mM NAC 60 minutes prior to the addition of CSE and LPS resulted in restoration of LPS-induced whole cellular CCR7 levels (Figure 4B), suggesting that rather than inhibiting translocation of preformed CCR7 to the surface of dendritic cells, CSE inhibits *de novo *formation of cellular CCR7 following LPS activation. To directly determine whether CSE suppresses migration of dendritic cells, immature and LPS-activated human dendritic cells were incubated with or without CSE for 18 hours. Half a million dendritic cells under different conditions were washed and resuspended in 0.5 ml RPMI without serum and placed in cell culture inserts. The inserts were subsequently placed in a 12-well tissue culture plate containing 0.5 ml of RPMI with 10% fetal calf serum and 10 ng/ml of CCL21, the respective ligand for CCR7. After 4 hours of incubation in a tissue culture chamber, the number of cells that migrated from the insert to the lower chamber was quantified. As expected, few immature dendritic cells migrated from the upper chamber to the lower chamber, while dendritic cells matured with LPS demonstrated a marked increase in migratory capacity (1745 ± 6.3 vs 10665 ± 106.1 cells; p < 0.001 with 1-way ANOVA and Bonferroni's Multiple Comparison Test). Surprisingly, dendritic cells activated with LPS in the presence of CSE did not demonstrate a reduction in migratory capacity (Figure 4C). In contrast, migration of LPS-activated dendritic cells conditioned with CSE towards the CCR7 ligand was augmented (Figure 4C; 10665 ± 106.1 vs 14385 ± 551.5 cells for LPS-activated dendritic cells and CSE-conditioned LPS-activated dendritic cells respectively; p < 0.001 with 1-way ANOVA and Bonferroni's Multiple Comparison Test). Although CSE did not diminish migration of LPS-activated dendritic cells towards CCL21, a statistically significant reduction in migration was observed in LPS-activated dendritic cells conditioned with 1000 ng/ml nicotine (Figure 4C; 10665 ± 106.1 vs 8205 ± 275.8 cells for LPS-activated dendritic cells and nicotine conditioned LPS-activated dendritic cells respectively; p < 0.01 with 1-way ANOVA and Bonferroni's Multiple Comparison Test).

## Discussion

Recent studies have demonstrated abnormalities in adaptive immunity against specific antigens in COPD patients [26], and rekindled interest in the role of autoimmunity as a central mechanism in the pathogenesis of COPD [2]. Relatively little is know about the roles of dendritic cells in the pathogenesis of cigarette smoke-induced COPD. In the current study we utilized human COPD tissue specimens and other experimental models to provide evidence consistent with recently published studies reporting increased dendritic cell numbers in COPD [3]. We expand the observations reported in that study by showing that COPD is associated with a significant increase in the total number of CD83+ dendritic cells. We demonstrate that COPD lung tissue contains increased levels of the Langerhans cell restricted gene CD207, and that there is correlation between osteopontin and CD207 gene levels. The current study shows that CSE and cigarette smoke enhance human and murine dendritic cell survival respectively, and that CSE induces endogenous dendritic cell pro-survival factors. This study also shows that although activated dendritic cells conditioned with CSE have diminished migratory CCR7 expression - an effect induced by oxidative constituents in CSE - migration of CSE-conditioned activated dendritic cells towards the CCR7 ligand CCL21 is not impaired, implying that retention of activated dendritic cells due to impaired CCR7-dependent migration is unlikely to be an important mechanism for the increased dendritic cell numbers in COPD.

Virtually all lung compartments (conducting airways, interstitial and alveolar spaces, vascular structures, and pleura) contain dendritic cells [4]. Whereas, in mice, the integrin CD11c is a reasonable marker to identify murine dendritic cells, human lung dendritic cells are more challenging to characterize [4,27]. The trachea and large conducting airways have a well-developed network of intraepithelial dendritic cells which share many properties with skin Langerhans' cells, and express intracellular CD207 and surface CD1a [4]. Although some studies have suggested that cigarette smoke exposure is associated with a reduction in murine lung conventional dendritic cell populations [28,29], the majority of studies (including our own [30]) report expansion of murine lung dendritic cell numbers following chronic (generally defined as 4 weeks or greater) cigarette smoke exposure [30-35]. Increased numbers of lung dendritic cells were also reported to occur in SCID mice exposed to chronic cigarette smoke exposure [36]. Cigarette smoke also increases the number of lung dendritic cells following allergen challenge in murine lungs [31]. The current study provides additional evidence that COPD is associated with increased numbers of matured dendritic cells identifiable by the marker CD83. Our data are consistent with a recent report by Freeman et al, in which CD80 and CD83 expression on isolated lung dendritic cells were reported to increase with severity of COPD [37]. A limitation of the current study is a lack of a non-smoking group as a control, as all patients from whom lung tissue was procured where either current or former smokers at the time of lung biopsy. Nevertheless, the current study shows that in smokers (either current or former) with COPD, increased numbers of CD83+ cells and CD207 mRNA occurs, which cannot be explained simply by chronic exposure to tobacco since the cumulative exposure to tobacco in the two groups was not different. Another limitation of our study is that is does not definitively show that COPD is associated with increased numbers of either Langerhans type dendritic cells or matured myeloid dendritic cells, as it may be argued that an alternative explanation to our findings is that COPD is associated with altered expression of these receptors or dendritic cell markers, rather than an increase in the respective dendritic cell populations *per se*.

It is well recognized that cigarette smoke modulates immunity by altering the function of several immune cells, including dendritic cells [11,25,30,33,38]. Cigarette smoke constituents known to having immune altering effects include nicotine [39-43], carbon monoxide [44], acrolein [45], reactive oxidant species [46], peroxynitrites [47], and possibly others. In the current study we did not identify a specific cigarette smoke constituent responsible for altered dendritic cell viability or induction of pro-survival cellular proteins. The current study implicates reactive oxidative species [47,48], a term which broadly refers to a wide collection of chemicals present in cigarette smoke that have the capacity to increase cellular oxidative stress. In addition to oxidative stress, other cigarette smoke constituents may also be responsible for altered dendritic cell viability and retention in COPD. Although our data suggests that nicotine is not primarily responsible for enhanced dendritic cell survival *in vitro*, or augmentation of dendritic cell survival proteins, it does not completely rule out the possibility that nicotine may have additive or synergistic effects with other chemicals in cigarette smoke (including those that induce cellular oxidative stress) that may have a significant effect on dendritic cell survival and retention in the COPD lung.

Our findings differ with a recent report that showed a reduction in CD83+ dendritic cells in COPD tissues compared to controls [6]. In that study, mature dendritic cells defined as CD83+ cells, were identified by immunohistochemistry on 41 lung tissue samples from individuals with COPD [6]. In contrast to the findings described in the current report, those authors observed fewer numbers of CD83+ cells in the small airways of COPD tissue compared with control smokers without COPD and non-smoker controls [6]. The reasons for the discrepant findings are not clear. In the study by Tsoumakidou et al [6], all subjects had primary lung carcinoma. In the current study, 20 out of the 24 subjects (5 of the 8 controls and 17 of the 18 COPD samples) used in the immunohistochemical determination of CD83 had a diagnosis of either non-small cell (15) or small cell carcinoma (5). Although cancer itself is known to be associated with reduced numbers of mature dendritic cells, this is not likely to have been a source of bias, since our study showed greater CD83 staining in COPD tissue (virtually all of which had coexistent lung carcinoma) compared to controls. Another potential source of bias in our study is the lack of information regarding corticosteroid therapy in COPD patients included in the current analysis. However, this is highly unlikely to have significantly altered the conclusions of the study, since corticosteroid therapy has been associated with a reduction - rather than an increase - in a number of dendritic cells functions, including maturation [49-51].

The mechanisms by which smoking promotes dendritic cell recruitment or retention in the lung are not fully elucidated. The current study demonstrates that cigarette smoke components activate endogenous dendritic cell survival pathways. In this report, we focused on two key intermediary molecules that have previously been reported to have important roles in protecting cells from death by apoptosis [19]. We used an *in vitro *approach with CSE as a stimulant to determine whether cigarette smoke induces pro-survival factors in dendritic cells. This approach was utilized in favor of direct determination of protein levels in lung tissue dendritic cells. Dendritic cells are a rare population of cells, and quantitative determination of cellular protein levels in human lung tissue is extremely difficult to accomplish. Bcl-xL is a member of the Bcl-2 family of apoptosis regulators that control apoptotic cellular response to oxidants and other stressors [20,21]. Heme-oxygenase-1 is a cellular stress response protein shown to be induced by a variety of cellular stressors including cigarette smoke [52-55]. Incubation of human dendritic cells with CSE resulted in induction of HO-1 and Bcl-xL protein levels, and suggests that induction of pro-survival factors by CSE may be an important mechanism by which CSE promotes dendritic cell survival *in vitro*. Our data do not provide direct evidence that CSE-induced HO-1 or Bcl-xL promote dendritic cell survival. This proved impossible to test, as many of the techniques used to abrogate upregulation of these proteins (including siRNA or pharmacologic inhibitors), also resulted in diminished cellular survival due to cell toxicity or other non-specific effects.

Abnormalities in chemokine expression may also be responsible for excessive influx and delayed trafficking of dendritic cells. Demedts and colleagues identified CCR6 expression on dendritic cells and elevated CCL20 levels in sputum samples from COPD patients suggesting a potential interaction between dendritic cell CCR6 and epithelial CCL20 expression as a potential mechanism explaining the enhanced Langerhans cell numbers seen in COPD patients in their study [3]. Concordant with that observation, Brattke and colleagues showed that myeloid dendritic cells obtained by BAL from smokers expressed lower levels of surface CCR7, a chemokine receptor expressed by activated dendritic cells and required for migration out of the lung and homing to lymph nodes [25]. Those studies suggest the possibility that enhanced dendritic cell retention occurs in COPD due to epithelial-dendritic cell interactions and endogenous abnormalities in dendritic cell chemokine receptor expression. Our findings expand on those observations. We demonstrate that relative osteopontin gene transcript levels correlate highly with CD207 gene levels. Osteopontin is a secreted phosphoprotein potently induced in epithelial cells by CSE [13], that regulates a variety of dendritic cell functions ranging from survival, maturation, and migration. While our data does not prove any direct association between induction of the osteopontin gene and dendritic cell infiltration in COPD, it does indicate parallel induction of the osteopontin and CD207 genes occurs in smokers with COPD, and suggests the possibility that cigarette smoke-induced osteopontin levels in the lung may be involved in recruitment or formation of Langerhans type dendritic cells in smokers with COPD. In support of this contention is a recent study that reported an increase in pulmonary Langerhans cells and macrophages in murine lungs that over-expressed osteopontin following delivery of the human osteopontin gene by an adenoviral vector [16]. In the current report we also sought to determine whether the reduced CCR7 expression described to occur in human dendritic cells isolated by BAL from smokers [25], or observed in human dendritic cells conditioned by CSE *in vitro *[11], is associated with reduced migration. We initially hypothesized that CSE-conditioned dendritic cells would have diminished migration towards a CCR7 ligand. The current report demonstrates that although CSE suppresses CCR7 expression *in vitro*, there is no limitation in dendritic cell migration towards CCR7 ligands. Although contrary to our original hypothesis, this data is consistent with the report by Robbins et al that found no difference in the migration of ovalbumin-loaded lung dendritic cells - achieved by intra-tracheal delivery of FITC-tagged ovalbumin - to the regional lymph nodes in mice chronically exposed to cigarette smoke [29]. In addition to migration, the CCR7 receptor is responsible for a number of non-migratory dendritic cell functions that may be relevant to COPD pathogenesis. Thus, it is conceivable that although migration towards CCR7 ligands is intact in dendritic cells conditioned by CSE, other CCR7-dependent non-migratory dendritic cell functions in smokers may be significantly altered.

In conclusion, we have used a number of complimentary approaches using human COPD lung tissue, *in vitro *and *in vivo *murine models to demonstrate that COPD is associated with increased numbers of cells bearing the CD83 receptor and CD207, and that CSE and cigarette smoke enhance dendritic cell survival. Enhanced osteopontin levels and augmented dendritic cell pro-survival factors are potential mechanisms that mediate the increased dendritic cell numbers. Our data do not support reduced emigration due to suppressed CCR7 expression as a mechanism for dendritic cell retention in the lungs of smokers. Elucidating the roles of dendritic cells in COPD will be essential in the quest for understanding the pathogenesis and ultimately therapy of this frustrating disease.

## Abbreviations used

FEV1: forced expiratory volume in 1 second; FVC: forced vital capacity; COPD: chronic obstructive pulmonary disease; HO-1: heme-oxygenase-1; Bcl-xL: B cell lymphoma leukemia-x(L); NAC: N acetyl cysteine; RPMI: Roswell Park Memorial Institute media; LPS: lipopolysaccharide; RIN: RNA integrity number; LTRC: lung tissue research consortium

## Competing interests

The authors declare that they have no competing interests.

## Authors' contributions

RV obtained funding for experiments on murine and human dendritic cells, conducted experiments, analyzed data, generated figures, and wrote the manuscript. PRW designed and performed the murine and human dendritic cell experiments; collected and analyzed data. JL conducted all the experiments using human COPD tissue. TJK performed the PCR experiments described. ESY assisted with morphologic interpretation and immunohistochemical analysis. AHL obtaining funding for immunohistochemical tissue analysis, provided input on data analysis and interpretation, and helped prepare the final manuscript. All authors read and approved the final manuscript.

## References

[B1] Pauwels RA Rabe KF Burden and clinical features of chronic obstructive pulmonary disease (COPD) Lancet 2004 364 9434 613 620 10.1016/S0140-6736(04)16855-4 15313363

[B2] Cosio MG Saetta M Agusti A Immunologic aspects of chronic obstructive pulmonary disease N Engl J Med 2009 360 23 2445 2454 10.1056/NEJMra0804752 19494220

[B3] Demedts IK Bracke KR Van Pottelberge G Testelmans D Verleden GM Vermassen FE Joos GF Brusselle GG Accumulation of dendritic cells and increased CCL20 levels in the airways of patients with chronic obstructive pulmonary disease Am J Respir Crit Care Med 2007 175 10 998 1005 10.1164/rccm.200608-1113OC 17332482

[B4] Vermaelen K Pauwels R Pulmonary dendritic cells Am J Respir Crit Care Med 2005 172 5 530 551 10.1164/rccm.200410-1384SO 15879415

[B5] Tsoumakidou M Demedts IK Brusselle GG Jeffery PK Dendritic cells in chronic obstructive pulmonary disease: new players in an old game Am J Respir Crit Care Med 2008 177 11 1180 1186 10.1164/rccm.200711-1727PP 18337593

[B6] Tsoumakidou M Koutsopoulos AV Tzanakis N Dambaki K Tzortzaki E Zakynthinos S Jeffery PK Siafakas NM Decreased Small Airway and Alveolar CD83+ Dendritic Cells in COPD Chest 2009 19465512 10.1378/chest.08-2824

[B7] Rogers AV Adelroth E Hattotuwa K Dewar A Jeffery PK Bronchial mucosal dendritic cells in smokers and ex-smokers with COPD: an electron microscopic study Thorax 2008 63 2 108 114 10.1136/thx.2007.078253 17875567

[B8] Pauwels RA Buist AS Calverley PM Jenkins CR Hurd SS Global strategy for the diagnosis, management, and prevention of chronic obstructive pulmonary disease. NHLBI/WHO Global Initiative for Chronic Obstructive Lung Disease (GOLD) Workshop summary Am J Respir Crit Care Med 2001 163 5 1256 1276 11316667 10.1164/ajrccm.163.5.2101039

[B9] Rabe KF Hurd S Anzueto A Barnes PJ Buist SA Calverley P Fukuchi Y Jenkins C Rodriguez-Roisin R van Weel C Global strategy for the diagnosis, management, and prevention of chronic obstructive pulmonary disease: GOLD executive summary Am J Respir Crit Care Med 2007 176 6 532 555 10.1164/rccm.200703-456SO 17507545

[B10] Bongartz T Cantaert T Atkins SR Harle P Myers JL Turesson C Ryu JH Baeten D Matteson EL Citrullination in extra-articular manifestations of rheumatoid arthritis Rheumatology (Oxford) 2007 46 1 70 75 10.1093/rheumatology/kel202 16782731

[B11] Vassallo R Tamada K Lau JS Kroening PR Chen L Cigarette smoke extract suppresses human dendritic cell function leading to preferential induction of Th-2 priming J Immunol 2005 175 4 2684 2691 16081845 10.4049/jimmunol.175.4.2684

[B12] Kroening PR Barnes TW Pease L Limper A Kita H Vassallo R Cigarette smoke-induced oxidative stress suppresses generation of dendritic cell IL-12 and IL-23 through ERK-dependent pathways J Immunol 2008 181 2 1536 1547 18606709 10.4049/jimmunol.181.2.1536 PMC2819390

[B13] Parsanejad R Fields WR Steichen TJ Bombick BR Doolittle DJ Distinct regulatory profiles of interleukins and chemokines in response to cigarette smoke condensate in normal human bronchial epithelial (NHBE) cells J Interferon Cytokine Res 2008 28 12 703 712 10.1089/jir.2008.0139 18937544

[B14] Woodruff PG Koth LL Yang YH Rodriguez MW Favoreto S Dolganov GM Paquet AC Erle DJ A distinctive alveolar macrophage activation state induced by cigarette smoking Am J Respir Crit Care Med 2005 172 11 1383 1392 10.1164/rccm.200505-686OC 16166618 PMC2718436

[B15] Xanthou G Alissafi T Semitekolou M Simoes DC Economidou E Gaga M Lambrecht BN Lloyd CM Panoutsakopoulou V Osteopontin has a crucial role in allergic airway disease through regulation of dendritic cell subsets Nat Med 2007 13 5 570 578 10.1038/nm1580 17435770 PMC3384679

[B16] Prasse A Stahl M Schulz G Kayser G Wang L Ask K Yalcintepe J Kirschbaum A Bargagli E Zissel G Essential role of osteopontin in smoking-related interstitial lung diseases Am J Pathol 2009 174 5 1683 1691 10.2353/ajpath.2009.080689 19359522 PMC2671257

[B17] Jarvik ME Madsen DC Olmstead RE Iwamoto-Schaap PN Elins JL Benowitz NL Nicotine blood levels and subjective craving for cigarettes Pharmacol Biochem Behav 2000 66 3 553 558 10.1016/S0091-3057(00)00261-6 10899369

[B18] Henningfield JE Stapleton JM Benowitz NL Grayson RF London ED Higher levels of nicotine in arterial than in venous blood after cigarette smoking Drug Alcohol Depend 1993 33 1 23 29 10.1016/0376-8716(93)90030-T 8370337

[B19] Tomita K Caramori G Lim S Ito K Hanazawa T Oates T Chiselita I Jazrawi E Chung KF Barnes PJ Increased p21(CIP1/WAF1) and B cell lymphoma leukemia-x(L) expression and reduced apoptosis in alveolar macrophages from smokers Am J Respir Crit Care Med 2002 166 5 724 731 10.1164/rccm.2104010 12204872

[B20] Boise LH Gonzalez-Garcia M Postema CE Ding L Lindsten T Turka LA Mao X Nunez G Thompson CB bcl-x, a bcl-2-related gene that functions as a dominant regulator of apoptotic cell death Cell 1993 74 4 597 608 10.1016/0092-8674(93)90508-N 8358789

[B21] Hon H Rucker EB III Hennighausen L Jacob J bcl-xL is critical for dendritic cell survival in vivo J Immunol 2004 173 7 4425 4432 15383573 10.4049/jimmunol.173.7.4425

[B22] Forster R Schubel A Breitfeld D Kremmer E Renner-Muller I Wolf E Lipp M CCR7 coordinates the primary immune response by establishing functional microenvironments in secondary lymphoid organs Cell 1999 99 1 23 33 10.1016/S0092-8674(00)80059-8 10520991

[B23] Jang MH Sougawa N Tanaka T Hirata T Hiroi T Tohya K Guo Z Umemoto E Ebisuno Y Yang BG CCR7 is critically important for migration of dendritic cells in intestinal lamina propria to mesenteric lymph nodes J Immunol 2006 176 2 803 810 16393963 10.4049/jimmunol.176.2.803

[B24] Ohl L Mohaupt M Czeloth N Hintzen G Kiafard Z Zwirner J Blankenstein T Henning G Forster R CCR7 governs skin dendritic cell migration under inflammatory and steady-state conditions Immunity 2004 21 2 279 288 10.1016/j.immuni.2004.06.014 15308107

[B25] Bratke K Klug M Bier A Julius P Kuepper M Virchow JC Lommatzsch M Function-associated surface molecules on airway dendritic cells in cigarette smokers Am J Respir Cell Mol Biol 2008 38 6 655 660 10.1165/rcmb.2007-0400OC 18203971

[B26] Lee SH Goswami S Grudo A Song LZ Bandi V Goodnight-White S Green L Hacken-Bitar J Huh J Bakaeen F Antielastin autoimmunity in tobacco smoking-induced emphysema Nat Med 2007 13 5 567 569 10.1038/nm1583 17450149

[B27] Jung S Unutmaz D Wong P Sano G De los Santos K Sparwasser T Wu S Vuthoori S Ko K Zavala F In vivo depletion of CD11c(+) dendritic cells abrogates priming of CD8(+) T cells by exogenous cell-associated antigens Immunity 2002 17 2 211 220 10.1016/S1074-7613(02)00365-5 12196292 PMC3689299

[B28] Robbins CS Dawe DE Goncharova SI Pouladi MA Drannik AG Swirski FK Cox G Stampfli MR Cigarette smoke decreases pulmonary dendritic cells and impacts antiviral immune responsiveness Am J Respir Cell Mol Biol 2004 30 2 202 211 10.1165/rcmb.2003-0259OC 12920055

[B29] Robbins CS Franco F Mouded M Cernadas M Shapiro SD Cigarette smoke exposure impairs dendritic cell maturation and T cell proliferation in thoracic lymph nodes of mice J Immunol 2008 180 10 6623 6628 18453581 10.4049/jimmunol.180.10.6623 PMC2885874

[B30] Vassallo R Kroening PR Parambil J Kita H Nicotine and oxidative cigarette smoke constituents induce immune-modulatory and pro-inflammatory dendritic cell responses Mol Immunol 2008 45 12 3321 3329 10.1016/j.molimm.2008.04.014 18533267 PMC2857673

[B31] Van Hove CL Moerloose K Maes T Joos GF Tournoy KG Cigarette smoke enhances Th-2 driven airway inflammation and delays inhalational tolerance Respir Res 2008 9 42 10.1186/1465-9921-9-42 18489797 PMC2408577

[B32] Bracke KR D'Hulst AI Maes T Moerloose KB Demedts IK Lebecque S Joos GF Brusselle GG Cigarette smoke-induced pulmonary inflammation and emphysema are attenuated in CCR6-deficient mice J Immunol 2006 177 7 4350 4359 16982869 10.4049/jimmunol.177.7.4350

[B33] D'Hulst AI Vermaelen KY Brusselle GG Joos GF Pauwels RA Time course of cigarette smoke-induced pulmonary inflammation in mice Eur Respir J 2005 26 2 204 213 10.1183/09031936.05.00095204 16055867

[B34] Maes T Bracke KR Vermaelen KY Demedts IK Joos GF Pauwels RA Brusselle GG Murine TLR4 is implicated in cigarette smoke-induced pulmonary inflammation Int Arch Allergy Immunol 2006 141 4 354 368 10.1159/000095462 16940747

[B35] Botelho FM Gaschler GJ Kianpour S Zavitz CC Trimble NJ Nikota JK Bauer CM Stampfli MR Innate Immune Processes are Sufficient for Driving Cigarette Smoke Induced Inflammation in Mice Am J Respir Cell Mol Biol 2009 42 4 394 403 10.1165/rcmb.2008-0301OC 19502389

[B36] D'Hulst AI Maes T Bracke KR Demedts IK Tournoy KG Joos GF Brusselle GG Cigarette smoke-induced pulmonary emphysema in scid-mice. Is the acquired immune system required? Respir Res 2005 6 147 10.1186/1465-9921-6-147 16359546 PMC1334210

[B37] Freeman CM Martinez FJ Han MK Ames TM Chensue SW Todt JC Arenberg DA Meldrum CA Getty C McCloskey L Lung Dendritic Cell Expression of Maturation Molecules Increases with Worsening COPD Am J Respir Crit Care Med 2009 180 12 1179 88 10.1164/rccm.200904-0552OC 19729666 PMC2796731

[B38] Lu LM Zavitz CC Chen B Kianpour S Wan Y Stampfli MR Cigarette smoke impairs NK cell-dependent tumor immune surveillance J Immunol 2007 178 2 936 943 17202355 10.4049/jimmunol.178.2.936

[B39] Aicher A Heeschen C Mohaupt M Cooke JP Zeiher AM Dimmeler S Nicotine strongly activates dendritic cell-mediated adaptive immunity: potential role for progression of atherosclerotic lesions Circulation 2003 107 4 604 611 10.1161/01.CIR.0000047279.42427.6D 12566374

[B40] Mamata Y Hakki A Yamamoto Y Newton C Klein TW Pross S Friedman H Nicotine modulates cytokine production by Chlamydia pneumoniae infected human peripheral blood cells Int Immunopharmacol 2005 5 4 749 756 10.1016/j.intimp.2004.12.010 15710343

[B41] Matsunaga K Klein TW Friedman H Yamamoto Y Involvement of nicotinic acetylcholine receptors in suppression of antimicrobial activity and cytokine responses of alveolar macrophages to Legionella pneumophila infection by nicotine J Immunol 2001 167 11 6518 6524 11714820 10.4049/jimmunol.167.11.6518

[B42] Nouri-Shirazi M Guinet E Evidence for the immunosuppressive role of nicotine on human dendritic cell functions Immunology 2003 109 3 365 373 10.1046/j.1365-2567.2003.01655.x 12807482 PMC1782971

[B43] Zhang S Petro TM The effect of nicotine on murine CD4 T cell responses Int J Immunopharmacol 1996 18 8-9 467 478 10.1016/S0192-0561(96)00054-9 9023586

[B44] Hegazi RA Rao KN Mayle A Sepulveda AR Otterbein LE Plevy SE Carbon monoxide ameliorates chronic murine colitis through a heme oxygenase 1-dependent pathway J Exp Med 2005 202 12 1703 1713 10.1084/jem.20051047 16365149 PMC2212966

[B45] Lambert C McCue J Portas M Ouyang Y Li J Rosano TG Lazis A Freed BM Acrolein in cigarette smoke inhibits T-cell responses J Allergy Clin Immunol 2005 116 4 916 922 10.1016/j.jaci.2005.05.046 16210070

[B46] Khan N Rahim SS Boddupalli CS Ghousunnissa S Padma S Pathak N Thiagarajan D Hasnain SE Mukhopadhyay S Hydrogen peroxide inhibits IL-12 p40 induction in macrophages by inhibiting c-rel translocation to the nucleus through activation of calmodulin protein Blood 2006 107 4 1513 1520 10.1182/blood-2005-04-1707 16249388

[B47] Muller T Haussmann HJ Schepers G Evidence for peroxynitrite as an oxidative stress-inducing compound of aqueous cigarette smoke fractions Carcinogenesis 1997 18 2 295 301 10.1093/carcin/18.2.295 9054621

[B48] Pryor WA Cigarette smoke radicals and the role of free radicals in chemical carcinogenicity Environ Health Perspect 1997 105 Suppl 4 875 882 10.2307/3433297 9255574 PMC1470037

[B49] Moller GM Overbeek SE Van Helden-Meeuwsen CG Van Haarst JM Prens EP Mulder PG Postma DS Hoogsteden HC Increased numbers of dendritic cells in the bronchial mucosa of atopic asthmatic patients: downregulation by inhaled corticosteroids Clin Exp Allergy 1996 26 5 517 524 10.1111/j.1365-2222.1996.tb00571.x 8735863

[B50] Georas SN Inhaled glucocorticoids, lymphocytes, and dendritic cells in asthma and obstructive lung diseases Proc Am Thorac Soc 2004 1 3 215 221 10.1513/pats.200402-004MS 16113437

[B51] Verhoeven GT Van Haarst JM De Wit HJ Simons PJ Hoogsteden HC Drexhage HA Glucocorticoids hamper the ex vivo maturation of lung dendritic cells from their low autofluorescent precursors in the human bronchoalveolar lavage: decreases in allostimulatory capacity and expression of CD80 and CD86 Clin Exp Immunol 2000 122 2 232 240 10.1046/j.1365-2249.2000.01354.x 11091280 PMC1905776

[B52] Sexton K Balharry D BeruBe KA Genomic biomarkers of pulmonary exposure to tobacco smoke components Pharmacogenet Genomics 2008 18 10 853 860 10.1097/FPC.0b013e328307bddf 18794723

[B53] Almolki A Guenegou A Golda S Boyer L Benallaoua M Amara N Bachoual R Martin C Rannou F Lanone S Heme oxygenase-1 prevents airway mucus hypersecretion induced by cigarette smoke in rodents and humans Am J Pathol 2008 173 4 981 992 10.2353/ajpath.2008.070863 18787101 PMC2543067

[B54] Kim HP Wang X Chen ZH Lee SJ Huang MH Wang Y Ryter SW Choi AM Autophagic proteins regulate cigarette smoke-induced apoptosis: protective role of heme oxygenase-1 Autophagy 2008 4 7 887 895 18769149 10.4161/auto.6767

[B55] Baglole CJ Sime PJ Phipps RP Cigarette smoke-induced expression of heme oxygenase-1 in human lung fibroblasts is regulated by intracellular glutathione Am J Physiol Lung Cell Mol Physiol 2008 295 4 L624 636 10.1152/ajplung.90215.2008 18689604 PMC2575948

